# A methodology to abridge microdosimetric distributions without a significant loss of the spectral information needed for the RBE computation in carbon ion therapy

**DOI:** 10.1002/acm2.14049

**Published:** 2023-05-25

**Authors:** Alessio Parisi, Chris J. Beltran, Keith M. Furutani

**Affiliations:** ^1^ Department of Radiation Oncology Mayo Clinic Jacksonville Florida USA

**Keywords:** AMDM, clonogenic survival, MCF MKM, microdosimetry, particle therapy, PHITS, relative biological effectiveness

## Abstract

**Background:**

In order to compute the relative biological effectiveness (RBE) of ion radiation therapy with the Mayo Clinic Florida microdosimetric kinetic model (MCF MKM), it is necessary to process entire microdosimetric distributions. Therefore, a posteriori RBE recalculations (i.e., for a different cell line or another biological endpoint) would require whole spectral information. It is currently not practical to compute and store all this data for each clinical voxel.

**Purpose:**

To develop a methodology that allows to store a limited amount of physical information without losing accuracy in the RBE calculations nor the possibility of a posteriori RBE recalculations.

**Methods:**

Computer simulations for four monoenergetic ^12^C ion beams and a ^12^C ion spread‐out Bragg peak (SOBP) were performed to assess lineal energy distributions as a function of the depth within a water phantom. These distributions were used in combination with the MCF MKM to compute the in vitro clonogenic survival RBE for human salivary gland tumor cells (HSG cell line) and human skin fibroblasts (NB1RGB cell line). The RBE values were also calculated with a new abridged microdosimetric distribution methodology (AMDM) and compared with the reference RBE calculations using the entire distributions.

**Results:**

The maximum relative deviation between the RBE values computed using the entire distributions and the AMDM was 0.61% (monoenergetic beams) and 0.49% (SOBP) for the HSG cell line, while 0.45% (monoenergetic beams) and 0.26% (SOBP) for the NB1RGB cell line.

**Conclusion:**

The excellent agreement between the RBE values computed using the entire lineal energy distributions and the AMDM represents a milestone for the clinical implementation of the MCF MKM.

## 1 INTRODUCTION

Most of the clinical experience of ion radiation therapy has been with protons and carbon ions.^1^ Carbon ions, due to their higher linear energy transfer (LET), exhibit significant variability in their relative biological effectiveness (RBE). Validated biophysical models are used during treatment planning to account for these RBE changes.^2^

Due to the large number of voxels in a real patient plan, it is currently impractical to store in each voxel detailed radiation quality information (for instance microdosimetric spectra or the particle‐specific fluence distributions of the kinetic‐energy and/or the LET) necessary for the computation of the RBE with different models, for different cell lines, and biological endpoints. Consequently, it is general practice to compute and store in each voxel the results of cell‐, endpoint‐, and model‐specific calculations.^3^, ^4^ However, this approach does not allow a fast and accurate a posteriori recalculation of the RBE for a different case.

Mayo Clinic Florida (MCF) is currently building the first‐in‐America combined proton and carbon ion therapy center in Jacksonville, Florida. Aiming to overcome some limitations of previous microdosimetric kinetic models (MKMs),^5^ we developed a new MKM model named MCF MKM.^6^ The MCF MKM predictions were validated against published in vitro and in silico clonogenic survival data for human and rodent cell lines exposed to ions from ^1^H to ^238^U.^6^, ^7^, ^8^ The MCF MKM formalism requires the knowledge of the entire microdosimetric spectrum for the RBE calculations. In order to include the microdosimetric events for the carbon ions and the secondary particles, the lineal energy distributions are generally computed over 6 lineal energy decades (10^−2^‐10^4^ keV/μm) using 50 bins per decade (300 bins in total).^6^ This is far exceeding the amount of data that can be currently stored in each clinical voxel.

Thus, we report the development and the first results of a methodology to summarize the microdosimetric distributions without losing a significant amount of spectral information nor hindering the possibility of RBE calculations for different cell lines.

## 2 METHODOLOGY

In order to benchmark the proposed methodology (**paragraph 2.3**), we performed radiation transport simulations (**paragraph 2.1**) and computed the lineal energy distributions as a function of the depth in water for four monoenergetic ^12^C ion beams and a ^12^C ion spread‐out Bragg peak (SOBP). In a second step, we coupled the lineal energy distributions with the MCF MKM^6^ (**paragraph 2.2**) to assess the RBE for two different cell lines. The RBE was calculated processing the entire lineal energy spectrum (reference calculations) and using the abridged microdosimetric distribution methodology (AMDM) described in **paragraph 2.3**. The results were then compared.

The clonogenic cell survival was chosen as the relevant biological endpoint for the calculation of the RBE.^2^, ^3^, ^4^ The linear quadratic model (LQM)^9^ was used to describe the relationship between the cell surviving fraction (*S*) and the absorbed dose (*D*), as in Equation (1)

(1) $$S\;=\;exp\left(-\alpha D-\beta D^{2}\right)$$

where α and β are the linear and quadratic terms of the LQM, respectively.

The RBE was evaluated as a function of the cell surviving fraction *S* with Equation (2)

(2) $$RBE_{S}=\;\frac{\alpha \;+\;\sqrt{\alpha^{2}-\;4\;\beta \;\ln\left(S\right)}}{\alpha_{ref}\;+\;\sqrt{\alpha_{ref}^{2}\;-\;4\;\beta_{ref}\;\ln\left(S\right)}}$$

where α, β, α_ref_, and β_ref_ are the linear and quadratic terms of the LQM for the radiation under investigation (carbon ions) and the reference photons, respectively.

In this article, we present only the results for the low dose RBE (RBE_α_) since the largest variations in the RBE are observed at low doses. As a result, RBE_α_ shows the largest differences between the reference RBE (calculated by processing the entire linear energy spectrum) and the AMDM‐based RBE. Therefore, this represents the worst‐case scenario for our methodology, as illustrated in Figure S1 in the Supplementary Materials. The low‐dose RBE (RBE_α_) was calculated with Equation (3) as the ratio between the linear terms of the LQM for carbon ions (α) and the reference photons (α_ref_). Equation (3) can be derived from Equation (2) as the RBE in the limit of *S*→1.

(3) $$\mathrm{RB}E_{\alpha }=\;\frac{\alpha }{\alpha_{\mathrm{ref}}}$$

In this work, the reference radiation was chosen to be 6 MV x‐rays.

### 2.1 Computer simulations

The Particle and Heavy Ion Transport code System (PHITS^10^) version 3.28 was used for all radiation transport simulations. A simulated water phantom of outer dimensions of 50 × 50 × 350 mm^3^ (density = 1 g/cm^3^) was irradiated with a point mono‐directional beam (radius = 0) of ^12^C ions impinging orthogonally on the center of one of the square surfaces. Four monoenergetic ^12^C ion beams were simulated: 100 MeV/n (minimum ^12^C ion energy of the upcoming accelerator at MCF), 220 MeV/n, 330 MeV/n, and 430 MeV/n (maximum ^12^C ion energy of the upcoming accelerator at MCF). No initial energy spread was used to create narrow Bragg peaks (worst case scenario for the methodology of **paragraph 2.3**). Additionally, to test the methodology in a clinically relevant scenario, we used a multi‐energy ^12^C beam source to generate a SOBP whose integrated depth dose profile is similar to the 350 MeV/n ^12^C SOBP used at the National Institute of Radiological Sciences (NIRS, Chiba, Japan) for the definition of the reference conditions in the clinical‐dose scaling process.^11^

The PHITS‐implementation of the Electron Gamma Shower version 5 (EGS5) code^12^ was used to transport photons, electrons, and positrons. The macroscopic energy loss of the other charged particles was simulated with the stopping power model ATIMA^13^ under the continuous slowing down approximation. The event generator mode version 2^14^ was used for the transport and interaction of low energy neutrons. The simulation cutoff energies for the radiation‐transport were set to 1 keV/n for all ions and to 1 keV for the other particles except neutrons (neutron cutoff = 10^−11^ eV). As recommended by the International Commission on Radiation Units and Measurements (ICRU),^15^ the mean excitation energy of liquid water was set to 78 eV. The energy straggling of charged particles was considered with the Landau‐Vavilov formula. The angular straggling was assessed with the Lynch's Coulomb diffusion formula based on Moliere's theory. The default nuclear reaction models of PHITS were used.^10^

To include the contribution of most secondary particles, the lineal energy distributions were scored for all particles using the PHITS microdosimetric function^16^ within phantom slices of dimensions 50 × 50 × 1 mm^3^. The lineal energy spectra were computed for water spheres with a radius of 0.30 μm. This value is in line with the size of the subnuclear domains listed in Table 1. The RBE_α_ calculated using lineal energy distributions for spheres with radius equal to the cell‐specific values of 0.28 μm (HSG cell line^6^) and 0.32 μm (NB1RGB cell line^7^) would be equivalent. The minimum energy deposition event considered in the calculations was that for one single ionization event, corresponding to a lineal energy of 0.027 keV/μm. Six lineal energy decades (10^−2^‐10^4^ keV/μm) with 50 bins per decade were used.

**TABLE 1:** MCF MKM parameters used for the HSG ^6^ and the NB1RGB^8^ cell lines: α_0_ (α in the limit of *y → 0*), β_0_ (β in the limit of *y → 0*), α_0_/β_0_ (indication of the cell radiosensitivity), R_n_ (mean radius of the cell nucleus), and r_d_ (mean radius of the subnuclear domains), α_ref_ (simulated α for the reference 6 MV x‐rays), β_ref_ (simulated β for the reference 6 MV x‐rays), α_ref_/β_ref_ (classical indication of the cell radiosensitivity).

Cell line abbreviation	α_0_ [Gy^−1^]	β_0_ [Gy^−2^]	$\frac{\alpha_{0}}{\beta_{0}}$ [Gy]	*R*_n_ [μm]	*r*_d_ [μm]	$\alpha_{ref}$ [Gy^−1^]	$\beta_{ref}$ [Gy^−1^]	$\frac{\alpha_{ref}}{\beta_{ref}}$ [Gy]
HSG	0.188	0.0572	3.29	4.5	0.28	0.273	0.0572	4.77
NB1RGB	0.390	0.0457	8.53	5.1	0.32	0.441	0.0457	9.67

[Correction added on 2 June, 2023, after first online publication: The values in the HSG row of columns 4 and 9 in Table 1 have been corrected.]

The calculations were performed on the mForge cluster of the National Center for Supercomputing Applications (NCSA, Urbana, Illinois) using 128 processor threads in parallel. In all cases (monoenergetic beams and SOBP), 10^7^ primary particles were simulated. The wall‐clock time of the simulations ranged between ∼7 and ∼22 h. Figures S2–S5 in the Supplementary Materials show that the choice of nuclear models and the number of simulated particles did not affect the agreement between the RBE values calculated using the AMDM and those calculated using the entire energy spectrum.

### 2.2 The MCF MKM

The linear term (α) of the LQM after irradiation with carbon ions was computed with the MCF MKM.^6^

At first, α was calculated as a function of the lineal energy *y* as in Equation (4)^6^

(4) $$\alpha \left(y\right)\;=\;\left(\alpha_{0}+\;\beta_{0}\frac{y}{\rho \;\pi \;r_{d}^{2}}\;\right)\left\{\frac{1-\exp\left[-\left(\alpha_{0\;}+\;\beta_{0}\;\frac{\;y}{\rho \;\pi \;r_{d}^{2}}\;\right)\;\frac{y}{\rho \;\pi \;R_{n}^{2}}\;-\;\beta_{0}\;{\left(\frac{y}{\rho \;\pi \;R_{n}^{2}}\right)}^{2}\;\right]}{\left(\alpha_{0\;}+\;\beta_{0}\;\frac{\;y}{\rho \;\pi \;r_{d}^{2}}\;\right)\;\frac{y}{\rho \;\pi \;R_{n}^{2}}\;+\;\beta_{0\;}\;{\left(\frac{y}{\rho \;\pi \;R_{n}^{2}}\right)}^{2}}\right\}$$

where, α_0_ and β_0_ are the LQM terms in the limit of *y → 0*, $R_{n}$ is the mean radius of the cell nucleus, *r*_d_ is the mean radius of the subnuclear domains, and *ρ* is the density (= 1 g/cm^3^). Equation (4) was derived^6^ from the formalism of the non‐Poisson MKM.^17^

In a second step, the simulated dose‐density distribution of the lineal energy *d(y)* in each computational subdomain (the computational phantom slices of **paragraph 2.1**) was used in combination with the cell‐specific α(*y*) (Equation 4) to compute α with Equation (5).^6^ The α values calculated with this approach were named α_spectrum_ to distinguish them from the ones obtained by using the AMDM (**paragraph 2.3**).

(5) $$\alpha_{spectrum}=\;\int \alpha \left(y\right)\;d\left(y\right)\;dy\;$$

It should be mentioned that, differently from other MKMs,^5^ the MCF MKM also includes an expression to describe the decrease of β at high LET.^6^, ^7^, ^8^

The MCF MKM relies on novel strategies^6^, ^7^ based on morphological and karyotypical measurements to determine a priori the cell‐specific values of *R*_n_ and *r*_d_. Consequently, the only in vitro data needed for the ion RBE calculations is the dose‐response of the cell after the reference photons. α_0_ is calculated knowing α_ref_ and the type of photons used for the reference irradiation.^6^, ^7^ In this work, the MCF MKM predictions were performed for the human salivary gland tumor cells (HSG cell) and normal skin fibroblasts (NB1RGB cell line). These cell lines were chosen for their clinical relevance and their different radiosensitivity. The validated^6^, ^7^ MCF MKM parameters are summarized in Table 1. For the NB1RGB cell line, these parameters are meant to be representative of the average radiosensitivity of the cell line over the different published experiments.^7^ For consistency between the two cell lines, the α_ref_ values needed for the calculation of RBE_α_ were simulated for the same reference radiation, namely 6 MV x‐rays. These α_ref_ values differ from the ones experimentally assessed using other photon qualities (200 kVp for the HSG cell line and different photon qualities for the NB1RGB cell line). Finally, RBE_α_ is computed dividing α by α_ref_.

### 2.3 The AMDM

In this work, we present a methodology to calculate relevant microdosimetric quantities over different linear energy ranges (named “bins”). These physical quantities can be subsequently used in conjunction with a microdosimetric model to compute the desired biological endpoint. Specifically, the calculations in this article are limited to the RBE_α_ computed with the MCF MKM.

At first, the microdosimetric spectrum is divided into *n* bins. *y*_min, bin_ *_j_* and *y*_max, bin_ *_j_* are the lower and upper lineal energy bounds of the bin *j*, respectively. The dose‐mean lineal energy in the bin *j*, named ${\bar{y}}_{D,\;bin\;j}$, is computed as in Equation (6)

(6) $${\bar{y}}_{D,\;bin\;j}=\;\;\frac{\int_{y_{min,\;bin\;j}}^{y_{max,\;bin\;j}\;}y\;d\left(y\right)dy}{\int_{y_{min,\;bin\;j}}^{y_{max,\;bin\;j}\;}d\left(y\right)dy}$$

where *d(y)* is the dose density distribution of the lineal energy *y*.

The fraction of absorbed dose deposited by lineal energy events within the bin *j* is named $\Delta_{bin\;j}$ and it is calculated with Equation (7).

(7) $$\Delta_{bin\;j}=\;\;\int_{y_{min,\;bin\;j}}^{y_{max,\;bin\;j}\;}d\left(y\right)dy$$

The cell‐specific linear term of the LQM in the bin *j* ($\alpha_{bin\;j}$, Equation 8) is calculated as the biological effect relative to the dose‐mean value of the lineal energy in each bin. This is mathematically done by substituting *y* in α(*y*) (Equation 4) with the ${\bar{y}}_{D,\;bin\;j}$ calculated with Equation (6).

(8) $$\alpha_{bin\;j}=\;\left(\alpha_{0}+\;\beta_{0}\;\frac{{\bar{y}}_{D_{bin,\;j}}}{\rho \;\pi \;r_{d}^{2}}\;\right)\left(\frac{1-\exp\left[-\left(\alpha_{0}+\;\beta_{0}\;\frac{{\bar{y}}_{D_{bin,\;j}}}{\rho \;\pi \;r_{d}^{2}}\;\right)\;\frac{{\bar{y}}_{D_{bin,\;j}}}{\rho \;\pi \;R_{n}^{2}}\;-\;\beta_{0}\;{\left(\frac{{\bar{y}}_{D_{bin,\;j}}}{\rho \;\pi \;R_{n}^{2}}\right)}^{2}\;\right]}{\left(\alpha_{0}+\;\beta_{0}\;\frac{{\bar{y}}_{D_{bin,\;j}}}{\rho \;\pi \;r_{d}^{2}}\;\right)\;\frac{{\bar{y}}_{D_{bin,\;j}}}{\rho \;\pi \;R_{n}^{2}}\;+\;\beta_{0}\;{\left(\frac{{\bar{y}}_{D_{bin,\;j}}}{\rho \;\pi \;R_{n}^{2}}\right)}^{2}}\right)$$

Finally, the results of the different bins are combined as in Equation (9) to calculate the cell‐specific value of the linear term of the LQM (named α_AMDM_).

(9) $$\alpha_{AMDM}=\;\;\sum_{j}\alpha_{bin\;j}\;\Delta_{bin\;j}$$

It is postulated that, using a limited amount of lineal energy bins, α_AMDM_ would be a reasonable approximation of the α calculated processing the entire spectrum (α_spectrum_ in Equation 5). In this work, we used 10 lineal energy bins.

## 3 RESULTS

**FIGURE 1:**
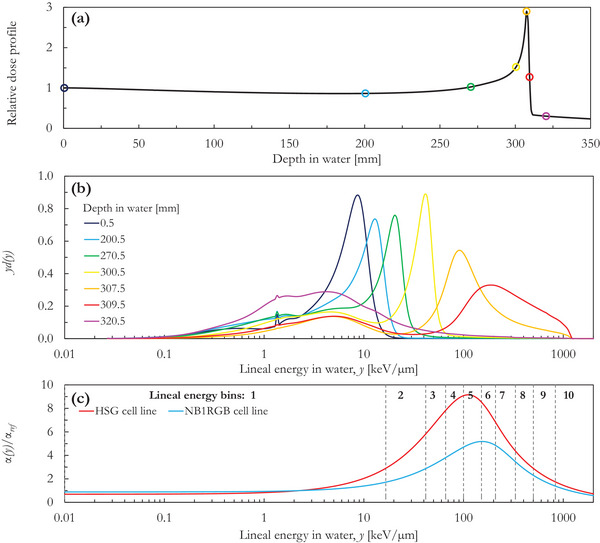
Relative integrated depth dose profile (a) and lineal energy distributions (b) simulated in water along the Bragg peak of a monoenergetic 430 MeV/n ^12^C ion beam. The relative absorbed dose profile was scored within 50 × 50 × 1 mm^3^ phantom slices. The lineal energy distributions plotted in (b) were assessed for liquid water spheres with a radius of 0.30 μm homogenously distributed within the phantom slices indicated by the circles in (a) with corresponding color. (c) The lineal energy dependence of α(y)/α_ref_ as calculated with the MCF MKM for the HSG and the NB1RGB cell lines, and the lineal energy bins used in the AMDM.

As an example of the simulated microdosimetric spectra, Figure 1 shows the lineal energy dose distribution for selected depths in water along the Bragg peak of the monoenergetic 430 MeV/n ^12^C ion beam. The circles in Figure 1a indicate the center of the phantom slices (50 × 50 × 1 mm^3^) where the microdosimetric spectra of Figure 1b were assessed. The distributions were computed for homogenously distributed water spheres with a radius equal to 0.3 μm. The α(*y*)/α_ref_ calculated with the MCF MKM is plotted in Figure 1c as a function of the lineal energy for both cell lines included in this study. The maximum value of the α(*y*)/α_ref_ functions depends on the cell line, being equal to 9.2 and 5.2 for the HSG and the NB1RGB cell lines, respectively. Furthermore, the position of the maximum also differs: 112 keV/μm for the HSG cell line and 155 keV/μm for the NB1RGB cell line. Nonetheless, both α(*y*)/α_ref_ functions tend to 0 for *y* → +∞. Figure 1c also shows the bins used in the AMDM. The bins were heuristically evaluated to minimize the risk of placing a bin in a region where the α(*y*)/α_ref_ function is not monotonic. To achieve this, narrower bins were employed over the lineal energy interval (50–500 keV/μm) where the peak of the cell specific α(*y*)/α_ref_ functions was expected to be found.

While most energy deposition events at the entrance plateau (depths in water between 0 and 200 mm) are characterized by relatively low lineal energy (< 20 keV/μm), a shift to much higher lineal energy values is observed at the distal edge. This is due to the slowing down of the carbon beam, resulting in higher LET. The carbon edge around ∼1000 keV/μm is clearly discernible in the spectrum for the phantom slice centered at 309.5 mm. The contribution of primary ^12^C ions is no longer visible in the spectrum in the fragmentation tail.

**FIGURE 2:**
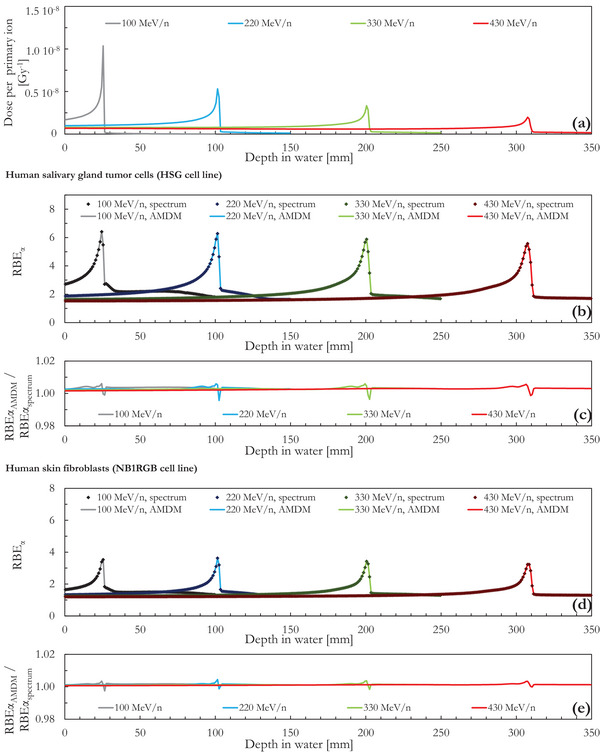
(a) Integrated depth dose profiles per unit of primary ion within 50 × 50 × 1 mm^3^ phantom slices for four monoenergetic ^12^C ions beams. (b) and (d) Comparison between the RBE_α_ values calculated with the MCF MKM using the AMDM (RBE_α,__AMDM_) and the entire microdosimetric distributions (RBE_α,spectrum_) for the HSG and the NB1RGB cell lines, respectively. (c) and (e) Ratio between the RBE_α, AMDM_ and the RBE_α,spectrum_ for the HSG and the NB1RGB cell lines, respectively.

The results of the four monoenergetic simulations are summarized in Figure 2. The integrated depth dose profiles plotted in Figure 2a serve as an indication of the relative position of the computed RBE values as a function of the depth in water. The plots of the fragmentation tails for the lower energy ^12^C ion beams are truncated to minimize data overlap. The results are expressed as the laterally integrated absorbed dose profile (the absorbed dose scored within the 50 × 50 × 1 mm^3^ phantom slices) per simulated primary particle. The absorbed dose includes the contributions of secondary particles. The RBE_α_ relative to 6 MV x‐rays is plotted in Figures 2b and 2d for the HSG and the NB1RGB cell lines, respectively. The RBE trends are similar for the four monoenergetic ions and are characterized by a monotonic increase up to a maximum value (RBE_α_ ∼6 and ∼3.5 for the HSG and the NB1RGB cell lines, respectively) followed by a sharp decrease. As quantified in Figures 2c and 2e, a very good agreement is present between the RBE values calculated with the MCF MKM by processing the entire lineal distributions (RBE_α,spectrum_) and the ones obtained with the abridged microdosimetric distribution methodology (RBE_α,AMDM_). The average ratio between the plotted RBE_α,AMDM_ and the RBE_α,spectrum_ values is 1.003 for the HSG cell line and 1.001 for the NB1RGB cell line. The maximum relative deviation between the two RBE_α_ data series is 0.61% for the HSG cell line and 0.45% for the NB1RGB cell line.

**FIGURE 3:**
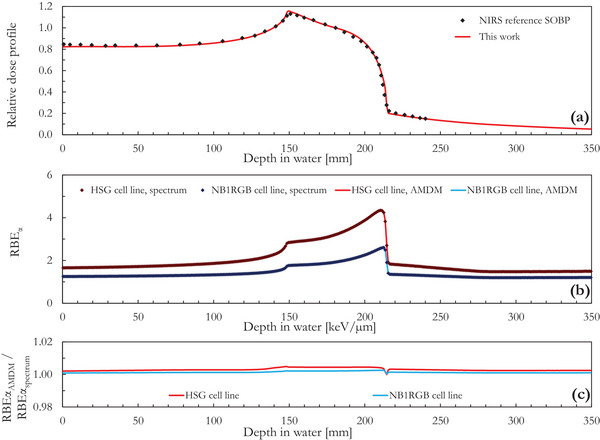
(a) Simplified simulation of a ^12^C ion SOBP in comparison with the NIRS reference SOBP.^10^ The relative integrated depth dose profiles were normalized to the center of the SOBP. (b) Comparison between the RBE_α_ values calculated with the MCF MKM using the AMDM (RBE_α, AMDM_) and the entire microdosimetric distributions (RBE_α, spectrum_) for the HSG and the NB1RGB cell lines. (c) Ratio between the RBE_α, AMDM_ and the RBE_α, spectrum_ for the HSG and the NB1RGB cell lines.

As example of a more clinically relevant scenario, Figure 3a presents the results of a SOBP whose integrated depth dose profile (Figure 3a) is qualitatively similar to that of the 350 MeV/n SOBP used at NIRS for the definition of the reference conditions for the clinical dose scaling.^11^ The dose profiles were normalized to the center of the SOBP (depth in water ∼181 mm). With respect to the monoenergetic beams of Figure 2, the increase of the RBE_α_ in Figure 3b is less sharp and the calculated RBE_α_ values are generally lower. As an example, the RBE_α_ at the center of the SOBP is 3.2 for the HSG cell line and 1.9 for the NB1RGB cell line. A good agreement was found between RBE_α,AMDM_ and RBE_α,spectrum_ values, with an average ratio (Figure 3c) of 1.003 for the HSG cell line and 1.001 for the NB1RGB cell line. The maximum relative deviation between the two RBE_α_ data series is 0.49% for the HSG cell line and 0.26% for the NB1RGB cell line.

## 4 DISCUSSION

The microscopic pattern of energy deposition of therapeutic carbon ions and secondary particles strongly varies along the primary particle path. As an example, the lineal energy distributions of Figure 1b, assessed at a scale relevant for modeling the radiation‐induced cell killing along a 430 MeV/n ^12^C ion Bragg peak, significantly differ between each other in value and shape. These distributions can be used in combination with microdosimetric models such as the MCF MKM^6^ to compute the RBE for different cell lines or endpoints. In this work, this was done by processing the obtained distributions with Equation (5) using the cell‐specific weighting functions shown in Figure 1c. This approach (processing entire microdosimetric distributions with the MCF MKM) was proven to provide accurate RBE predictions for very different exposure conditions and cell lines.^6^, ^7^, ^8^ However, its clinical application is hindered by the relatively large amount of data (300 bins) to be stored in each voxel (i.e., 1 × 1 × 1 mm^3^) for a realistic patient scenario. To overcome this issue, precalculated RBE values or linear‐quadratic terms for specific cell lines are generally used in the calculations.^3^, ^4^, ^11^ This approach is not computationally demanding but prevents an a posteriori RBE recalculation for a different cell line or another endpoint and forces the user to perform again the radiation transport simulations. With this in mind, we developed a new approach (AMDM) able to radically summarize the distributions without substantially losing spectral information.

By storing relevant physical information in a limited number of bins (i.e., 10 as done in this study) it was possible to compute the low dose RBE (RBE_α_) for two clinically relevant cell lines (HSG and NB1RGB) and five ^12^C ion exposure scenarios (four monoenergetic beams and a SOBP). A very good agreement was found between the AMDM calculations and the corresponding RBE values obtained processing the entire microdosimetric distributions (Figures 2 and 3, maximum deviation = 0.61% and 0.45% for the HSG and the NB1RGB cell lines, respectively). In this work, we presented the AMDM results for the RBE_α_ only since the largest RBE variations are observed at low dose. This is the worst‐case scenario for the AMDM. In other words, the maximum deviation between AMDM and the reference calculations is smaller at higher dose (Figure S1 of the Supplementary Materials).

It is worth noting that the AMDM differs from simply binning the spectrum over different energy ranges for the RBE calculations. In the latter case, the events are first scored within predefined lineal energy bins and, in a second step, the energy deposited by these events is associated to a bin‐specific lineal energy value, typically the centroid of the bin. If this approach were used to calculate the RBE_α_ (using for instance the same bins of Figure 1), the results would significantly differ from that of the reference calculations (maximum deviation equal to 39% and 18% for the HSG and the NB1RGB cell lines, respectively). In contrast, with the AMDM, the energy deposition from different events in the different lineal energy bins is associated with the dose‐mean lineal energy of those events in each bin ${\bar{y}}_{D,\;bin\;j}$.

In this work we used the AMDM along with the MCF MKM. Still, the AMDM could be used in conjunction with other microdosimetric models such as the clinically‐implemented modified MKM.^18^ This flexibility eliminates the need to pre‐select the cell line of interest or the microdosimetric model. In other words, using the AMDM, it becomes possible to store physical information that would allow for an a posteriori RBE calculation for any arbitrary cell line, endpoint, and microdosimetric model. This approach can be particularly useful for conducting retrospective analyses of previous treatment plans.

## 5 CONCLUSIONS

The RBE_α_ of the HSG and the NB1RGB cell lines was calculated with the MCF MKM for four monoenergetic ^12^C ion beams and a ^12^C ion SOBP. The RBE_α_ was computed by processing the entire microdosimetric spectra (6 lineal energy decades with 50 bins per decade, serving as reference calculations) and utilizing the new AMDM (10 lineal energy bins). The AMDM accurately reproduced the reference calculations with a maximum deviation of 0.6%, thus paving the way for the clinical implementation of the MCF MKM.

## AUTHOR CONTRIBUTIONS

Methodology: Alessio Parisi, Chris J. Beltran, and Keith M. Furutani; Software: Alessio Parisi; Formal analysis: Alessio Parisi; Visualization: Alessio Parisi; Writing original draft: Alessio Parisi; Writing review and editing: Alessio Parisi, Chris J. Beltran, and Keith M. Furutani; Supervision: Chris J. Beltran and Keith M. Furutani; Funding acquisition: Chris J. Beltran and Keith M. Furutani. All authors have read and agreed to the published version of the manuscript.

## CONFLICT OF INTEREST STATEMENT

The authors declare no conflict of interest.

## Supporting information

Supporting Information

## Data Availability

The data that support the findings of this study are available from the corresponding author upon reasonable request.

## References

[1] Durante M , Debus J , Loeffler JS . Physics and biomedical challenges of cancer therapy with accelerated heavy ions. Nat Rev Phys. 2021;3(12):777‐790. 34870097 10.1038/s42254-021-00368-5 PMC7612063

[2] Karger CP , Peschke P . RBE and related modeling in carbon‐ion therapy. Phys Med Biol. 2017;63(1):01TR02. 10.1088/1361-6560/aa9102 28976361

[3] Inaniwa T , Furukawa T , Kase Y , et al. Treatment planning for a scanned carbon beam with a modified microdosimetric kinetic model. Phys Med Biol. 2010;55(22):6721‐6737. 21030747 10.1088/0031-9155/55/22/008

[4] Mairani A , Brons S , Cerutti F , et al. The FLUKA Monte Carlo code coupled with the local effect model for biological calculations in carbon ion therapy. Phys Med Biol. 2010;55(15):4273‐4289. 20647603 10.1088/0031-9155/55/15/006

[5] Parisi A , Furutani KM , Beltran CJ . On the calculation of the relative biological effectiveness of ion radiation therapy using a biological weighting function, the microdosimetric kinetic model (MKM) and subsequent corrections (non‐Poisson MKM and modified MKM). Phys Med Biol. 2022;67(9):095014. 10.1088/1361-6560/ac5fdf 35474177

[6] Parisi A , Beltran CJ , Furutani KM . The Mayo Clinic Florida microdosimetric kinetic model of clonogenic survival: formalism and first benchmark against in vitro and in silico data. Phys Med Biol. 2022;67:185013. 10.1088/1361-6560/ac7375 36097336

[7] Parisi A , Beltran CJ , Furutani KM . The Mayo Clinic Florida microdosimetric kinetic model of clonogenic survival: application to various repair‐competent rodent and human cell lines. Int J Mol Sci. 2022;23(20):12491. 36293348 10.3390/ijms232012491 PMC9604502

[8] Parisi A , Beltran CJ , Furutani KM . Clonogenic survival RBE calculations in carbon ion therapy: the importance of the absolute values of α and β in the photon dose‐response curve and a strategy to mitigate their anticorrelation. Quantum Beam Sci. 2023;7(1):3.

[9] McMahon SJ . The linear quadratic model: usage, interpretation and challenges. Phys Med Biol. 2018;64(1):01TR01. 10.1088/1361-6560/aaf26a 30523903

[10] Sato T , Iwamoto Y , Hashimoto S , et al. Features of particle and heavy ion transport code system (PHITS) version 3.02. J Nucl Sci Technol. 2018;55(6):684‐690.

[11] Inaniwa T , Kanematsu N , Matsufuji N , et al. Reformulation of a clinical‐dose system for carbon‐ion radiotherapy treatment planning at the National Institute of Radiological Sciences, Japan. Phys Med Biol. 2015;60(8):3271‐3286. 25826534 10.1088/0031-9155/60/8/3271

[12] Hirayama H , Namito Y , Bielajew AF , Wilderman SJ , Nelson WR . SLAC‐R‐730 The EGS5 Code System. Stanford Linear Accelerator Center. 2005

[13] https://web‐docs.gsi.de/~weick/atima/

[14] Iwamoto Y , Niita K , Sato T , et al. Application and validation of event generator in the PHITS code for the low‐energy neutron‐induced reactions. Prog Nucl Sci Technol. 2011;2:931‐935.

[15] International Commission on Radiation Units and Measurements . ICRU Report No. 85: Fundamental quantities and units for ionizing radiation (revisited). 2011.

[16] Sato T , Watanabe R , Niita K . Development of a calculation method for estimating specific energy distribution in complex radiation fields. Radiat Prot Dosim. 2006;122(1‐4):41‐45. 10.1093/rpd/ncl407 17132656

[17] Hawkins RB . A microdosimetric‐kinetic model for the effect of non‐Poisson distribution of lethal lesions on the variation of RBE with LET. Radiat Res. 2003;160(1):61‐69. 12816524 10.1667/rr3010

[18] Kase Y , Kanai T , Matsumoto Y , et al. Microdosimetric measurements and estimation of human cell survival for heavy‐ion beams. Radiat Res. 2006;166(4):629‐638. 17007551 10.1667/RR0536.1

