# Supplementary material for: A methodology to abridge microdosimetric distributions without a significant loss of the spectral information needed for the RBE computation in carbon ion therapy
Source: J Appl Clin Med Phys. 2023 May 25;24(7):e14049. doi: 10.1002/acm2.14049 (PMC10338778; doi:10.1002/acm2.14049)
Supplement: Supplementary file 1 — Supporting Information [file ACM2-24-e14049-s001.docx]

# SUPPLEMENTARY MATERIALS

**SM1. Effect of the RBE endpoint on the agreement between AMDM and reference calculations**

In the main body of the article the results were presented for the low dose RBE (RBE_α_) since the largest deviations between the AMDM and the reference calculations (using the entire microdosimetric distributions) were for this RBE endpoint. This happens because the largest variations in the RBE occur at low dose.

**Figure SM1** show the effect of the chosen surviving fraction on the RBE calculations along the ^12^C ion SOBP for human salivary gland tumor cells (HSG cell line). The laterally integrated dose profile simulated with PHITS is plotted in **Figure SM1A**. As shown in **Figure SM1B**, lower RBE values were computed at lower surviving fraction. Additionally, the agreement between AMDM and reference calculations was better at lower surviving fraction (**Figure SM1C**). The max deviation between AMDM and reference calculations was 0.49% for RBE_α_, 0.45% for RBE_50%_, 0.39% for RBE_10%_, and 0.36% for RBE_1%_.

The simulations parameters are the same as in the main body of the article. A simulated water phantom of outer dimensions of 50x50x350 mm^3^ (density = 1 g/cm^3^) was irradiated with a pencil beam (radius = 0) of ^12^C ions impinging orthogonally on the center of one of the square surfaces. The absorbed dose was laterally integrated over 50x50x1 mm^3^ phantom slices. The lineal energy distributions were assessed for water spheres (radius = 0.30 µm) homogenously distributed within the phantom slices. 10^7^ primary particles were simulated.

The MCF MKM parameters for the HSG cell line were the same as in the in the article and are listed in **Table SM1**.

**Table SM1**. MCF MKM parameters used for the HSG cell line: α_0_ (α in the limit of *y 🡪 0*), *ß*_0_ (*ß* in the limit of *y 🡪 0*), *R*_n_ (mean radius of the cell nucleus), and *r*_d_ (mean radius of the subnuclear domains), α_ref_ (simulated α for the reference 6 MV X-rays), and *ß*_ref_ (simulated *ß* for the reference 6 MV X-rays).

| **Cell line abbreviation** | $\boldsymbol{\alpha}_{\boldsymbol{0}}$  **[Gy^-1^]** | $\boldsymbol{\beta}_{\boldsymbol{0}}$  **[Gy^-2^]** | ***R*_n_**  **[µm]** | ***r*_d_**  **[µm]** | $\boldsymbol{\alpha}_{\boldsymbol{ref}}$  **[Gy^-1^]** | $\boldsymbol{\beta}_{\boldsymbol{ref}}$  **[Gy^-1^]** |
| --- | --- | --- | --- | --- | --- | --- |
| HSG | 0.188 | 0.0572 | 4.5 | 0.28 | 0.273 | 0.0572 |

**
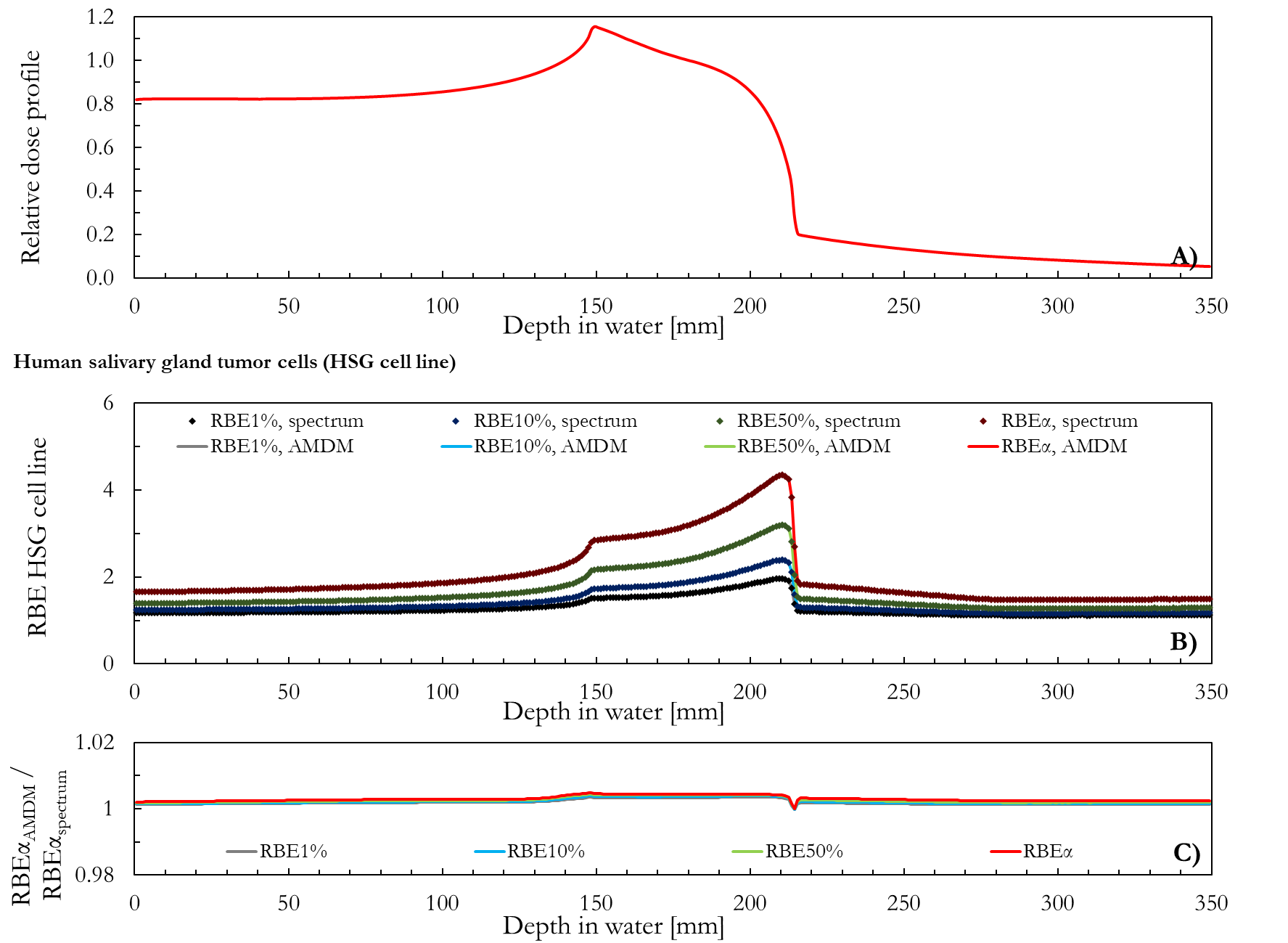
**

**Figure SM1. A**) Simplified simulation of the laterally integrated dose profile of a ^12^C ion SOBP. **B**) Comparison between the RBE values calculated with the MCF MKM using the entire microdosimetric distributions (“spectrum”) and the AMDM for the HSG cell line. **C**) Ratio between the RBE values calculated with the MCF MKM using the entire microdosimetric distributions (“spectrum”) and the AMDM for the HSG cell line.

**SM2. Effect of the number of simulated particles and the nuclear models on the agreement between AMDM and reference calculations**

This section shows the effect of the number of simulated particles and the nuclear fragmentation model on the MCF MKM RBE_α_ calculations for the HSG cell line.

At first, the RBE_α_ calculations for the ^12^C ion SOBP (physical and biological parameters described in **paragraph SM1**) were repeated varying the number of simulated primary particles from 10^7^ (reference calculations) to 10^4^, 10^5^, and 10^6^. The laterally integrated absorbed dose profile and the RBE_α_ for the HSG cell line were assessed as a function of the depth in water processing the entire microdosimetric distributions and utlizing the AMDM. The results of the calculations using the entire distributions and the AMDM were compared between each other and with the corresponding reference calculations for 10^7^ simulated primary particles.

The number of simulated particles was found to affect the integrated dose profile (panels A and B of **Figures SM2**, **SM3**, and **SM4**) and the RBE_α_ (panels C and D of **Figures SM2**, **SM3**, and **SM4**). Nonetheless, the agreement between the AMDM and the calculations using the entire microdosimetry distributions (panels E of **Figures SM2**, **SM3**, and **SM4**) was found to be unaffected by the number of simulated particles (maximum deviation = 0.49% regardless of the number of simulated primary particles).

Secondly, the calculations were repeated changing the nuclear fragmentation model used in the PHITS simulations (JQMD 2.0 instead of JQMD). JQMD is the default model in PHITS to describe the heavy ion interactions. JQMD 2.0 is a more recent, accurate, and computationally demanding version of JQMD (PHITS manual, <https://phits.jaea.go.jp/manual/manualE-phits.pdf>). The number of simulated primary particles (10^7^) was left unchanged as the other parameters. The results of the simulations for these two nuclear models are compared in **Figure SM5**. The choice of the nuclear model appears to significantly affect the results of the simulations in the fragmentation tail of the ^12^C ion SOBP, both with respect to the integrated absorbed dose (panels A and B of **Figures SM5**) and the RBE_α_ for the HSG cell line (panels C and D of **Figures SM2**). Nonetheless, as for the number of simulated primary particles, the choice of the nuclear fragmentation model does not affect the agreement between the RBE_α_ values computed processing the entire microdosimetric distributions and the AMDM-based ones (maximum deviation = 0.49% regardless of chosen nuclear model).

In conclusion, while both the absorbed dose and the RBE are influenced by the number of simulated particles and the nuclear reaction model selected, the results obtained using the AMDM and the entire microdosimetric distributions give the equivalent dependence on these variables as shown in panels E of **Figures SM2, SM3, SM4, and SM4.**


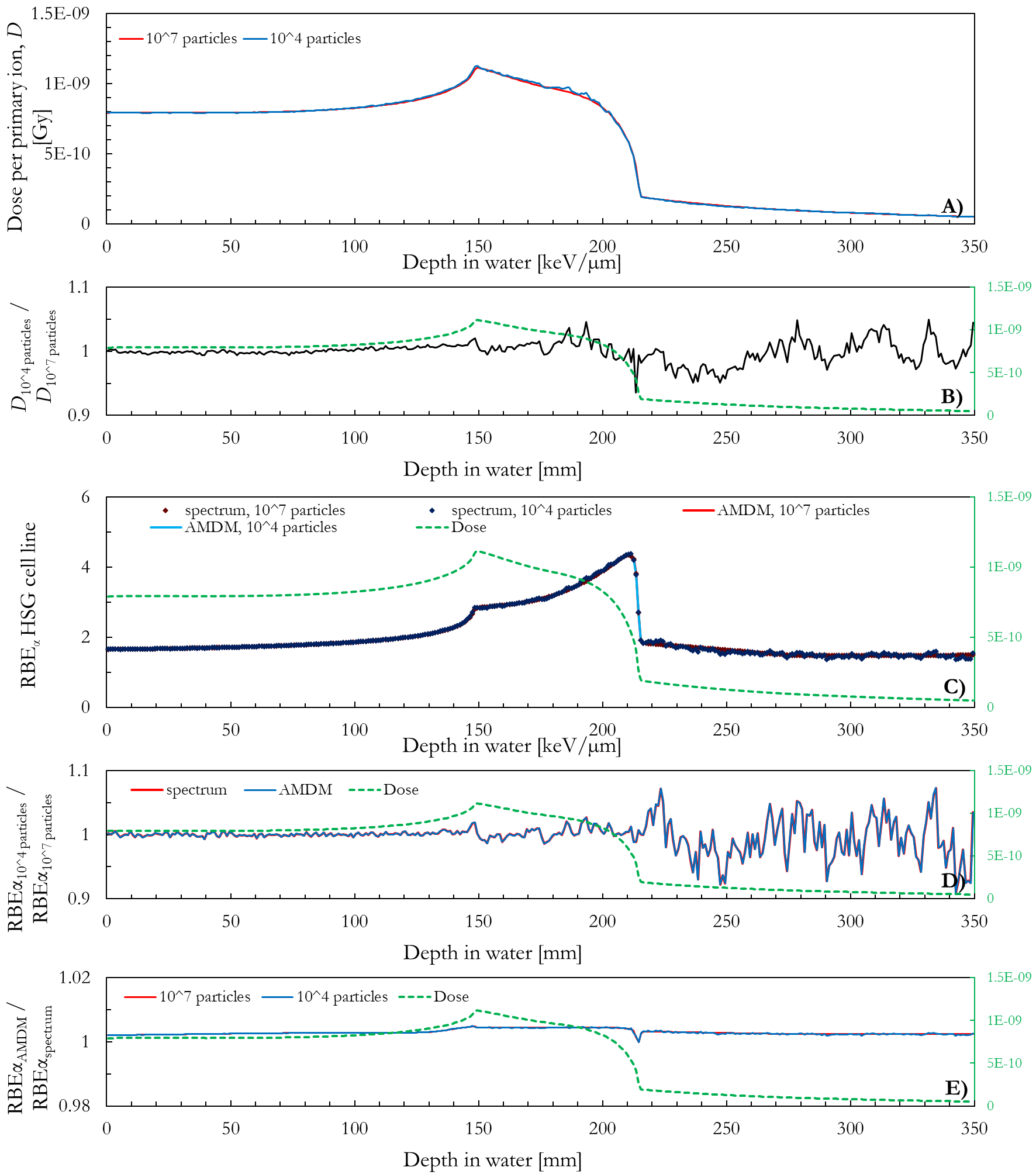


**Figure SM2.** Effect of the number of simulated primary particles (10^4^ vs reference calculations with 10^7^ particles) on the laterally integrated dose profile of a ^12^C ion SOBP (A and B) and the RBE_α_ of the HSG cell line calculated with the MCF MKM using the entire microdosimetric distributions and the AMDM (C, D, and E).

**
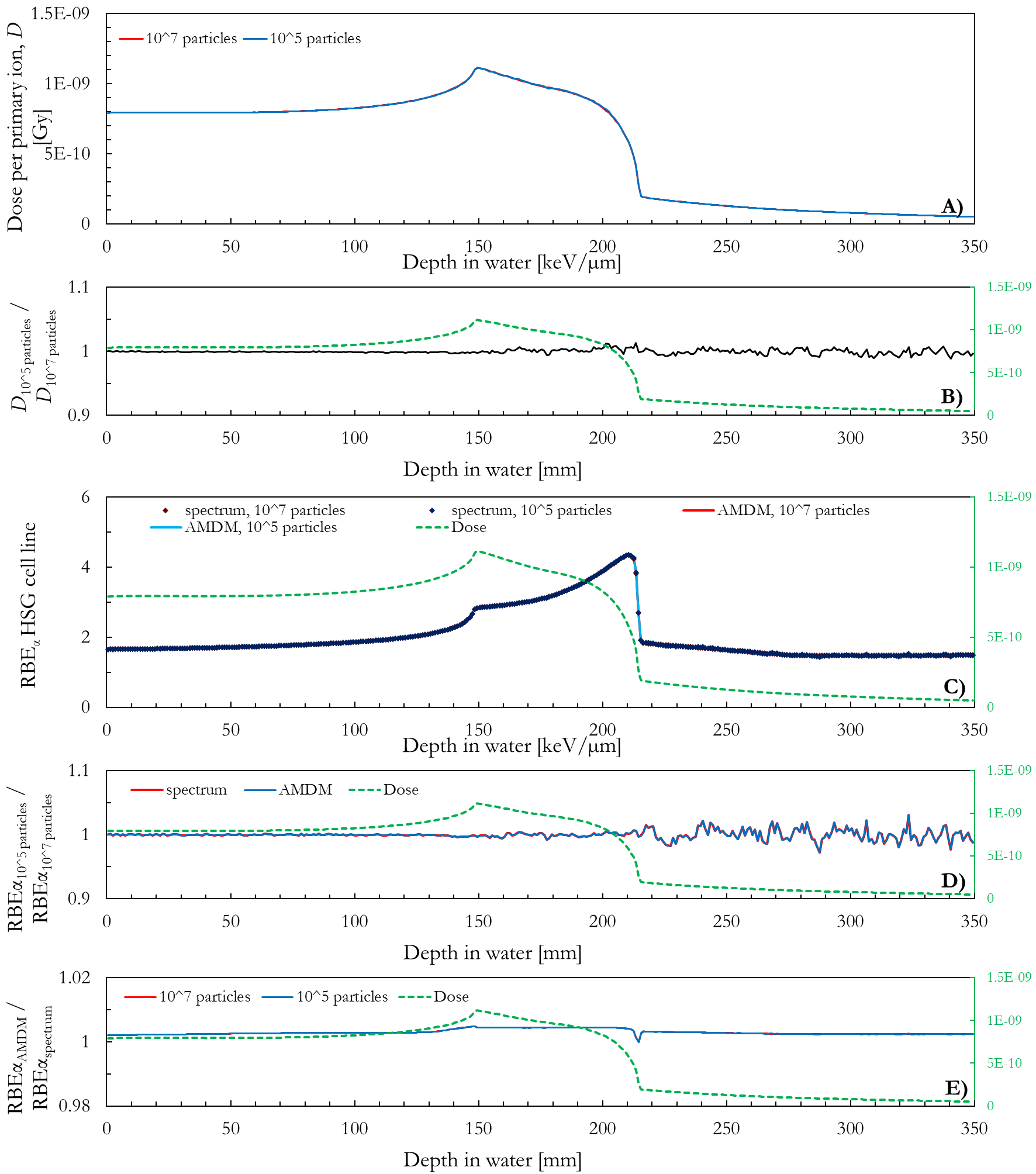
**

**Figure SM3.** Effect of the number of simulated primary particles (10^5^ vs reference calculations with 10^7^ particles) on the laterally integrated dose profile of a ^12^C ion SOBP (A and B) and the RBE_α_ of the HSG cell line calculated with the MCF MKM using the entire microdosimetric distributions and the AMDM (C, D, and E).

**
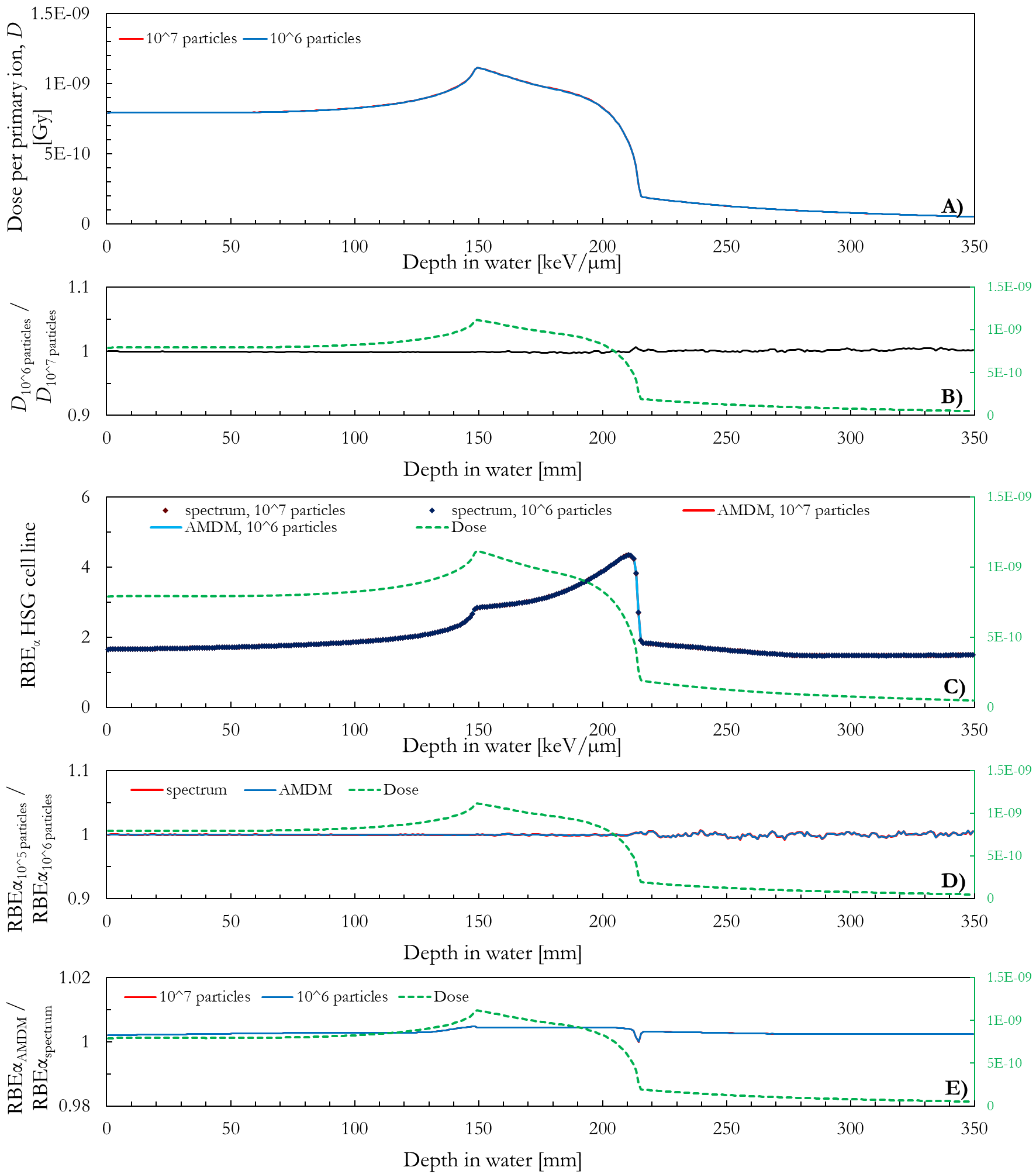
**

**Figure SM4.** Effect of the number of simulated primary particles (10^6^ vs reference calculations with 10^7^ particles) on the laterally integrated dose profile of a ^12^C ion SOBP (A and B) and the RBE_α_ of the HSG cell line calculated with the MCF MKM using the entire microdosimetric distributions and the AMDM (C, D, and E).

**
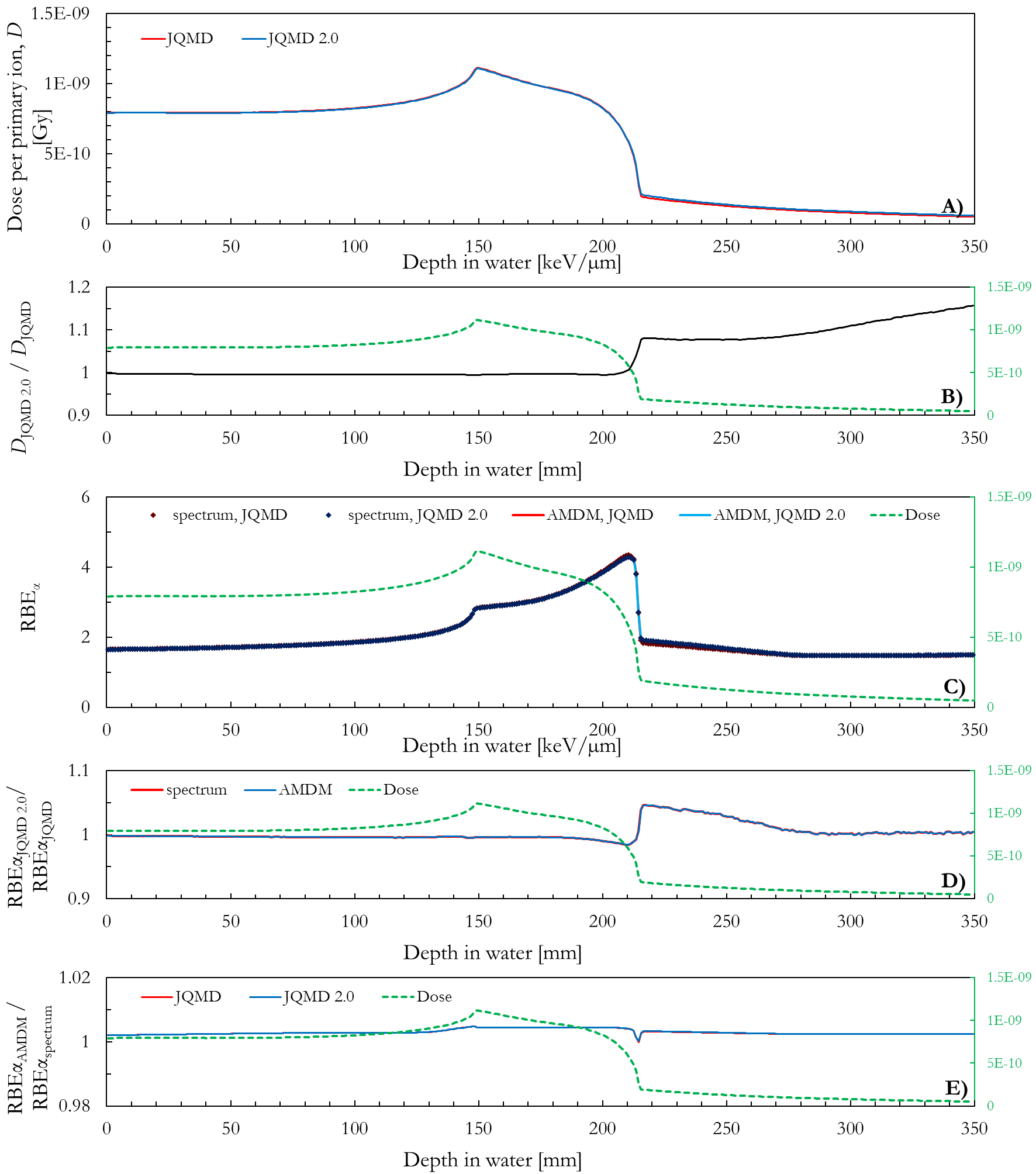
**

**Figure SM5.** Effect of the nuclear reaction model (JQMD 2.0 vs reference calculations with JQMD) on the laterally integrated dose profile of a ^12^C ion SOBP (A and B) and the RBE_α_ of the HSG cell line calculated with the MCF MKM using the entire microdosimetric distributions and the AMDM (C, D, and E).
